# Cancer Immunotherapy: A Focus on the Regulation of Immune Checkpoints

**DOI:** 10.3390/ijms19051389

**Published:** 2018-05-07

**Authors:** Tao Shi, Yanyu Ma, Lingfeng Yu, Jiaxuan Jiang, Sunan Shen, Yayi Hou, Tingting Wang

**Affiliations:** 1 The State Key Laboratory of Pharmaceutical Biotechnology, Division of Immunology, Medical School, Nanjing University, Nanjing 210093, China; 151230030@smail.nju.edu.cn (T.S.); 151230023@smail.nju.edu.cn (Y.M.); 151232054@smail.nju.edu.cn (L.Y.); 151230015@smail.nju.edu.cn (J.J.); shensn@nju.edu.cn (S.S.); yayihou@nju.edu.cn (Y.H.); 2 Jiangsu Key Laboratory of Molecular Medicine, Nanjing University, Nanjing 210093, China

**Keywords:** immune checkpoint regulation, personalized cancer immunotherapy, gut microbiota, oncolytic viruses, tumor microenvironment

## Abstract

In recent years, the role of cancer immunotherapy has become increasingly important compared to traditional cancer treatments, including surgery, chemotherapy and radiotherapy. Of note, the clinical successes of immune checkpoint blockade, such as PD-1 and CTLA-4, represent a landmark event in cancer immunotherapy development. Therefore, further exploration of how immune checkpoints are regulated in the tumor microenvironment will provide key insights into checkpoint blockade therapy. In this review, we discuss in details about the regulation of immune checkpoints mediated by immune cells, oncolytic viruses, epigenetics, and gut microbiota and mutual regulation by co-expressed checkpoints. Finally, predictions are made for future personalized cancer immunotherapy based on different checkpoint modulations.

## 1. Introduction

Cancer, a disease associated with cell growth caused by genetic mutations, is known to activate proto-oncogenes. This process is accompanied by the genetic or non-genetic activation or inactivation of specific genes that stimulate or inhibit the proliferation and metastasis of tumors. Though long considered promising, cancer immunotherapy has only been in the spotlight of tumor treatment over the last 3 to 5 years. It is generally regarded as the fourth tumor therapy after surgery, radiotherapy and chemotherapy. In addition, cancer immunotherapy was listed by Science as one of the top ten annual scientific breakthroughs in 2013 [1]. Of note, cancer immunotherapies targeting immune checkpoints have recently revolutionized cancer treatments. These treatments function through the blockade of immunosuppressive checkpoints, such as PD-1, CTLA-4, and TIGIT, and the activation of immunostimulatory checkpoints, such as CD226 and CD28, in effector T-cells and myeloid cells [2,3,4,5,6].

The human immune system comprises immune defense, immune surveillance and immune self-stabilization. The immune surveillance targets at transformed tumor cells in vivo for recognition and elimination. The antitumor immune effects of the body act through both cellular and humoral immune responses, with the function mediated mainly by cellular immunity. Cells that exert immune effects include T cells, NK cells, and macrophages, among which T cells (Th, Tc, Treg, etc.) play a vital role in cell-mediated immunity against cancers. The activation of T cells requires two signals: signal one is an antigen-specific signal to the T-cell receptor (TCR) by specific antigens on major histocompatibility (MHC) molecules that are expressed on antigen-presenting cells (APCs) or target cells; signal two is a co-stimulatory signal provided by B7 and other co-stimulatory molecules (B7-1/CD28, B7-2/CD28, B7-H2/ICOS, LFA-3/CD2, etc.) to assist in T cell activation. Co-inhibitory molecules (PD-1/PD-L1, PD-1/PD-L2, B7/CTLA-4, etc.) can function to hinder T cell signal transduction processes, thus restraining T-cell functions. The dynamic balance between co-stimulatory and co-inhibitory signals together determines T cell function or tolerance [7].

Immunosuppressive checkpoint blockade has achieved great breakthroughs over the last 3–5 years. Immunosuppressive checkpoints can inhibit immune responses mainly via interfernece with CD3/CD28-dependent signaling, competitive interruption of B7 family binding to co-stimulatory molecules or via transmission of supressive signals into tumor cells or T cells. Two of these checkpoints, namely, programmed cell death protein-1 (PD-1) and cytotoxic T-lymphocyte protein-4 (CTLA-4), have become the focus of this attention. CTLA-4 competitively inhibits the binding of the CD28 molecule to the B7 complex, hindering the function of the CD28 molecule of promoting T cell activation. CTLA-4 also interferes with TCR signaling by interacting with PP2A (protein phosphatase 2) and SHP2 (SH2 domain-containing protein-tyrosine phosphatase-2). Simultaneously, CTLA-4 binds to PI3K (phosphatidylinositide 3-kinase), leading to the phosphorylation of AKT (protein kinase B). The cell-surface receptor PD-1 is only expressed by T cells upon activation and binds to PD-L1 or PD-L2 to inhibit T cell activation. Ipilimumab (MDX-010, Yervoy; Bristol Myers Squibb, Princeton, NJ, USA), a fully human monoclonal antibody targeting CTLA-4 was approved by the U.S. Food and Drug Administration (FDA) for metastatic melanoma treatment in 2011 to improve survival [8]. On 22 December 2014, the FDA approved nivolumab (Opdivo; Bristol Myers Squibb, an antibody targets PD-1) to treat patients suffering from unresectable or metastatic melanoma [9]. Additionally, in 2015 Weber et al. found that patients treated with nivolumab achieved more objective responses and had fewer toxic effects than advanced melanoma patients who had progressed after ipilimumab or ipilimumab with a BRAF (proto-oncogene *B-Raf*) inhibitor [10]. Recently, other novel immune checkpoints have been under continuous research. First, T-cell immunoglobulin and ITIM (immunoreceptor tyrosine-based inhibitory motif) domain (TIGIT) is a co-inhibitory receptor that, together with the co-stimulatory receptor CD226, forms a pathway that is analogous to the CD28/CTLA-4 pathway. TIGIT can outcompete CD226 to bind with CD155 (PVR) and can interfere with the cis-homodimerization of CD226, thus delivering a cell-intrinsic inhibitory signal [4,11,12]. Second, V-domain Ig suppressor of T cell activation (VISTA) is highly expressed on hematopoietic cells within the TME (tumor micro-environment). Its blockade can lead to antitumor immunity enhancement in mice, though its mechanism of inhibiting T cell activation and its specific ligands are not yet clear [13]. Third, B7-H3 (CD276) has both co-stimulatory and co-inhibitory functions. As a co-stimulatory checkpoint, B7-H3 can increase the proliferation of CD4^+^ and CD8^+^ T cells and enhance the activity of cytotoxic T cells [14]. As a co-inhibitory checkpoint, B7-H3 correlates with tumor expansion, invasion, metastasis, and recurrence, and anti-B7-H3 antibodies can suppress cancer development both in vitro and in vivo [15,16].

Although a series of important immune checkpoints have been identified, the relationship between immune checkpoints, the degree of checkpoint expression and the functional roles of the checkpoints remain to be further explored. Additionally, multiple modulations of immune checkpoints have a deep impact on the efficacy of checkpoint blockade therapy. Therefore, we focused on different aspects of the regulation of immune checkpoints in the TME (tumor microenvironment), including regulation mediated by immune cells in the TME, oncolytic viruses, the function of epigenetic modulators, the role of intestinal flora and mutual regulation by various immune checkpoints. In the future, we expect to carry out more accurate and effective personalized cancer immunotherapy, after considering the different levels of checkpoint expression among patients based on the regulation mediated by different factors.

## 2. Regulation of Immune Checkpoints

### 2.1. Regulation Mediated by Myeloid and Lymphoid Cells in the Tumor Microenvironment

The tumor microenvironment (TME) is the cellular microenvironment where tumor cells and other components (including immune cells, fibroblasts, surrounding blood vessels, lymphoid cells, signaling molecules and the extracellular matrix) exist [17]. Within the TME, infiltrating myeloid cells (MDSC, TAM, TAN, etc.) and Tregs play important regulatory roles in immune checkpoints to promote tumor progression and modulate the function of tumor-infiltrating lymphocytes. Therefore, these cell types represent potential targets for cancer immunotherapy.

Myeloid-derived suppressor cells (MDSCs) are important cell components of the TME and include polymorphic nuclear MDSCs (PMN-MDSCs) and monocytic MDSCs (M-MDSCs). MDSCs mainly act to suppress immune cell activity. Here, we discuss the role of MDSCs in checkpoint regulation in the TME. Chemokines (CCL2, CCR2, CCL5, etc.) produced by different tumors can actively recruit MDSCs to primary and metastatic tumor sites to inhibit immune cell function within the TME [18,19]. The functions of MDSCs derived in the TME are different from the functions of those in peripheral lymphoid organs. Unresponsiveness to PD-1 or CTLA-4 blockade was associated with a dramatic accumulation of circulating MDSCs, and the continuous elimination of MDSCs by Ab depletion during PD-1 or CTLA-4 blockade treatment resulted in the almost complete eradication of established tumors [20]. Immune regulation of CTLA-4 and PD-1 by MDSCs within the TME may involve multiple mechanisms. First, hypoxia-inducible factor 1-α (HIF1-α)-mediated tumor-associated hypoxia increases PD-L1 expression on the surface of tumor-infiltrating MDSCs, thus resulting in the inhibition of T cell activity by binding to PD-1 expressed on T cells [21]. Second, studies report that the transmission of tumor-derived exosomes from cancer cells to MDSCs can increase PD-L1 expression on MDSCs in glioma and LLC tumor models. This phenomenon is likely to be related to TGF-β and IL-10 production by MDSCs [22]. Third, HIF1α can elevate Arg1 (Arginase-1) and iNOS (inducible nitric oxide synthase) expression, which is linked to the up-regulation of co-inhibitory checkpoint receptors and their ligands on MDSCs [23,24]. Lastly, the products of MDSCs, including TGF-β, IL-10, CCL4, CCL5, and ROS (reactive oxygen species), can activate other infiltrating myeloid cells and Tregs, thus up-regulating the expression of immune checkpoint molecules on these cells [25].

Tumor-associated macrophages (TAMs) and tumor-associated neutrophils (TANs) are also key components of the TME and participate in various aspects of tumor development. TAMs and TANs are derived from monocytic precursors that circulate in blood. Similar to MDSCs, TAMs and TANs are recruited to tumor sites by tumor-derived chemokines and growth factors, including CCL2/MCP-1, CXCL1/Gro-a, CCL7/MCP-3, CCL5/RANTES, CXCL8/IL-8, VEGF, PDGF, and M-CSF/CSF-1 [26]. The immunosuppressive function of TAMs and TANs is analogous to MDSCs and can up-regulate immune checkpoints and their receptors. Most recently, studies have shown that both mouse and human TAMs express PD-1 and that PD-1 expression increases gradually in mouse models. Thus, TAMs can directly interrupt T cell activation [27]. Additionally, PD-1^-^ TAMs can capture anti-PD-1 mAbs within minutes, which ought to be bound to PD-1 on T cell surfaces. The accuracy of anti-PD-1 mAbs is dependent both on the structure of the antibody’s Fc domain glycan and Fcγ receptors (FcγRs) expressed by TAMs [28]. Third, TANs express PD-1 and can increase the expression of cytokines, such as IL-17A, to mediate resistance to PD-1 blockade [29,30]. Lastly, similar to MDSCs, TAMs and TANs can produce cytokines and chemokines (TGF-β, IL-10, CCL22, B7-H1/H4, and ROS) to interact with MDSCs, Th, TC, Treg, DCs and NK cells, finally enhancing the inhibitory effects of immunosuppressive checkpoints.

Regarding lymphoid cells within the TME, T-regulatory cells (Tregs) play significant immunosuppressive roles and modulate the expression of immune checkpoint modulators. CD4^+^ Tregs are a highly immune suppressive subclass of CD4^+^ T cells, characterized by the expression of CD25 and FoxP3 (master regulators of Treg development and function) [31]. The generation of Tregs is induced by a variety of cytokines, including TGF-β, IL-10, and IL-35, and can be divided into multiple subtypes, such as CD4^+^CD25^+^Tr, Tr1, and Th3. Additionally, Tregs can regulate checkpoint functions through multiple mechanisms. First, it was found that co-inhibitory receptor CTLA-4 was constitutively expressed by Tregs, and B7 molecules were transmitted to the surface or inside of Tregs to bind to CTLA-4. Therefore, the maturation of antigen-presenting cells (APC), which should be promoted via the B7/CD28 pathway, is strongly blocked [32,33]. Second, Treg-specific CTLA-4 deficiency can be impaired through IL-2A consumption via CD25 [34]. Third, FoxP3 can directly down-regulate *IL-2* gene transcription and up-regulates *CTLA-4* and *IL-2RA* gene transcription [32]. Fourth, secretion of immune inhibitory cytokines (such as IL-10, TGF-β and IL-35) by Tregs can indirectly regulate the role of immune checkpoints in tumor development. Also, most recently, studies have reported that PD-1 or TIGIT-expressing Tregs have selective and higher immunosuppressive ability than Tregs without PD-1 or TIGIT expression in mice [35,36].

### 2.2. Regulation Mediated by Oncolytic Viruses

Oncolytic virus (OV) immunotherapy is a novel therapeutic method for cancer treatment that utilizes native or genetically modified viruses which can selectively replicate within tumor cells and induce acute immune responses in the TME. Recently, many viruses have been proposed as possible vectors for cancer treatment, including poliovirus, measles virus, adenoviruses, poxviruses, herpes simplex virus (HSV), coxsackieviruses, reovirus, Newcastle disease virus (NDV) and others (Table 1). T-VEC (a herpes virus encoded with GM-CSF (granulocyte-macrophage colony stimulating factor)) and H101 were approved in the US and China for melanoma and carcinoma treatment [37,38]. Oncolytic viruses regulate the role of immune checkpoints in the TME mainly by acting as genetic vectors to carry specific checkpoint antibodies and via the oncolysis and secretion of cytokines and chemokines to synergize with immune checkpoint inhibition.

On the one hand, genetically modified oncolytic viruses can directly secrete checkpoint antibodies against invading tumor cells. For example, attenuated measles virus (MV) vectors that encode CTLA-4 and PD-L1 antibodies can improve therapeutic outcomes in murine models of malignant melanoma and reduce tumor size [39]. Additionally, a novel recombinant myxoma virus (vPD-1), which can secrete a soluble form of PD-1 from infected cells, induces and maintains antitumor CD8^+^ T-cell responses and demonstrates safer and more effective outcomes than systemic αPD-1 antibodies [40]. It is of interest to see whether similar outcomes can be demonstrated with antibodies targeting immune checkpoints, such as LAG-3, TIM-3 and CD226, with oncolytic viruses. Furthermore, multiple studies have shown that combinations of oncolytic viruses and immune checkpoint blockade therapy have strong synergistic effects. First, OV-mediated immune activation (selective viral replication and directed induction of antitumor immune responses in the TME) is required for CD8^+^ T cells and NK cells to improve the effect of antibodies that interrupt PD-1/PD-L1- or CTLA4/B7-mediated immune suppression [41]. Second, oncolytic viruses encoding genes of specific proteins, such as GM-CSF, interferon-β (IFN-β), IL-2, IL-12, IL-15, and heat shock proteins (HSP), can dramatically enhance checkpoint blockade therapy. For example, SFV-IL-12 (a Semliki Forest virus-based vector encoding IL-12) can synergize with PD-1/PD-L1 blockade. Researchers found that SFV-IL-12 synergized with PD-1 blockade and tumor remission, and prolonged survival was shown in MC38 and bilateral B16-OVA mouse models [42]. Additionally, clinical studies showed that combinations of T-VEC with ipilimumab appeared to be more tolerable with greater efficacy than either T-VEC or ipilimumab therapy alone for stage IIIB-IV melanoma treatment. GM-CSF expression by T-VEC induced stronger antitumor immune responses and recruited higher numbers of mature macrophages and dendritic cells (DCs) into the TME [43].

However, oncolytic virus immunotherapy can also negatively modulate immune checkpoints. A study has confirmed that oncolysis mediated by viruses strongly induced the expression of PD-L1 in primary liver cancer and lung metastases. Dissemination of CD8^+^ T cells was completely inhibited in the combined treatment of viral infection and PD-1 blockade [44]. In addition, the SFV-IL-12 treatment mentioned above induced PD-L1 expression on the surface of cancer cells in an IFNγ-dependent pathway, which may explain the adaptive immune resistance mediated by PD-L1 [42]. Therefore, further evaluation of oncolytic virotherapy combined with checkpoint blockade therapy is strongly recommended in future clinical studies.

### 2.3. Regulation Mediated by Epigenetics

Epigenetic disorder plays a key role in tumor development. Epigenetic modulation is defined as DNA modifications that can transform chromatin structure and gene expression without any changes to the current nucleotide sequence [45]. DNA methylation and PTMs (post-translational histone modifications, including methylation, acetylation, ubiquitination and phosphorylation) are both epigenetic modulations. Additionally, the actions of non-coding RNAs (such as piRNAs, siRNAs, and microRNAs) that bind to mRNA can regulate the transcription and translation of the encoded RNA. Of note, recent studies on the function of epigenetic modulations in immune resistance and evasion have uncovered epigenetic modulators as key mechanisms to augment immune responses in the TME and restore immune surveillance and immune homeostasis. These findings provide a promising basis for studies employing combinations of epigenetic drugs and immune checkpoint blockade for cancer treatment. Here, we discuss several mechanisms through which epigenetic modulators can enhance immune responses to immune checkpoints within the TME.

First, histone modifications play an important role in immune checkpoint modulation, especially histone methylation and acetylation. Epigenetic modulators promoting histone acetylation can enhance the cell surface expression of immune checkpoint receptors or ligands. Although the up-regulation of immunosuppressive checkpoints may inhibit T cell activation, it greatly increases immune responses to checkpoint blockade therapy. Woods et al. revealed that HDACis (inhibitors targeting the epigenetic regulatory family of histone deacetylases) up-regulated PD-L1 and PD-L2 (to a lesser degree) in melanoma-bearing mice due to rapidly increased histone acetylation. Treatment combining HDACi and PD-1 blockade reduced tumor development and increased overall survival compared to HDACi or PD-1 blockade alone [46]. Additionally, histone acetylation inhibition, such as with entinostat, can reduce the MDSC population within the TME to augment checkpoint blockade therapies [20]. Furthermore, histone methylation can synergize with checkpoint blockade therapy. For example, H3K27me3 (trimethylation of lysine 27 on histone H3) is a key epigenetic modification for T cell differentiation. The presence of H3K27me3 on Foxp3 locus is necessary to maintain Treg functions while loss of H3K27me3 leads to increased Th-1 plasticity. A recent study showed that H3K27me3 inhibition combined with CTLA-4 blockade results in greater tumor remission and Treg reduction than CTLA-4 blockade therapy alone in melanoma-bearing mice. This finding indicates that H3K27me3 inhibition could improve checkpoint blockade therapy [47]. Moreover, HAT (Histone/protein acetyltransferases) is also required for the immune suppressive function of Foxp3^+^ Tregs. In 2013 Liu et al. found that the inhibition of one HAT named Ep300 (Ep300i) resulted in increased apoptosis and impaired cell suppressive function of Tregs and tumor growth was controlled in immunocompetent mice [48]. Therapeutic effects can be improved with combination of Ep300i and checkpoint blockade. Second, DNA methylation is tightly associated with checkpoint modulation. For example, epigenetic modulator AZA-Vidaza (DNA hypomethylating agent azacitidine) up-regulates PD-L1 expression in NSCLC (non-small cell lung cancer) cell lines. Researchers hypothesized that AZA combined with the blockade of PD-1 may augment immune responses in NSCLC models by turning immune inhibition into immune activation, especially in NSCLC models with low expression of PD-1/PD-L1 pathway modulators [49]. In 2015 two studies revealed for the first time that DNA methyltransferase inhibitors (DNMTis) can up-regulate immune signaling in ovarian cancer and colorectal cancer through endogenous viral mimicry pathway. DNMTis trigger cytosolic sensing of dsRNAs which are partly derived from endogenous retroviral elements, causing a type I interferon response and cancer cell apoptosis [50,51]. The signature expression of high viral defense in tumor cells could be associated with response to checkpoint blockade therapies [50]. Additionally, DNMTis can enhance responses to checkpoint blockade through up-regulation of certain chemokines that are expressed on T cells and increasing T cell infiltration into the TME. Peng et al. showed that DNMTi 5-aza-2′deoxycytidine (5AZAdC) can up-regulate tumor production of Th1-type chemokines CXCL9 and CXCL10 to increase effector T cell infiltration into TME and improve therapeutic response to PD-L1 blockade compared to PD-L1 blockade alone [52]. In addition, DNMT inhibitor 5-azacytidine in conjunction with CTLA-4 and PD-1 blockade therapy can selectively reduce MDSCs in mice bearing colorectal or metastatic breast cancer [20]. Although mechanisms indicating the specific reduction of MDSCs by DNA methylation have not been clear, treatment outcomes showed that cancers with immune resistance can be improved by combinations of DNA methylation with checkpoint blockade.

Third, the role of non-coding RNA in the regulation of immune checkpoints has become a new research hotspot. A study by Wei et al. in 2016 found that microRNA was a possible target for both *PD-1* and *CTLA-4* genes. They observed that miR-138 could bind the 3′-untranslated regions of both *CTLA-4* and *PD-1* genes to down-regulate CTLA-4 and PD-1 expression in CD4^+^ T cells, inhibiting the growth of intracranial glioma cells [53]. Additionally, microRNA can also modulate the expression of immune checkpoint ligands. Research by Chen et al. demonstrated that microRNA-200 (miR-200) can suppress the expression of PD-L1 on human mesenchymal lung cancer cell lines (H157, H1155, H1299 and H460) through automatic suppression of EMT and cancer metastasis [54]. Moreover, knockdown of PD-L1 or PD-L2 mediated by siRNA can enhance the expression of interferon-γ and antigen-specific CTLs, suggesting that siRNA is an attractive strategy for regulating PD-1 expression [55]. Although many non-coding RNAs can regulate the expression of immune checkpoint receptors or ligands, the precise regulation mechanism is not yet fully understood, which has led to difficulties in applying non-coding RNAs into clinical trials of immune checkpoint blockade.

### 2.4. Regulation Mediated by Gut Microbiota

More than 100 trillion microbes are harbored in the human gut and were recently found to be associated with checkpoint blockade therapy. Abundant evidence shows that alterations in gut microbiota composition are associated with a number of intricate diseases, among which cancer is the focus. Previous research has demonstrated that distinct microbiota or microbiota products can induce immune response alterations, including the induction of Tregs and Th17 cells [56]. Additionally, conventional chemotherapies, such as cyclophosphamide and oxaliplatin, are tightly linked to gut microbiota to enhance antitumor immune responses and achieve efficient tumor control [57]. Furthermore, the latest research reveals that the immune activity and antitumor effects of checkpoint blockade therapy are associated with distinct species of microbes in various tumor types. Here, we focus on two immune checkpoints (PD-1 and CTLA-4) and discuss how distinct gut-resident commensals modulate the efficacy of PD-L1 and CTLA-4 blockade therapies.

**Figure 1:**
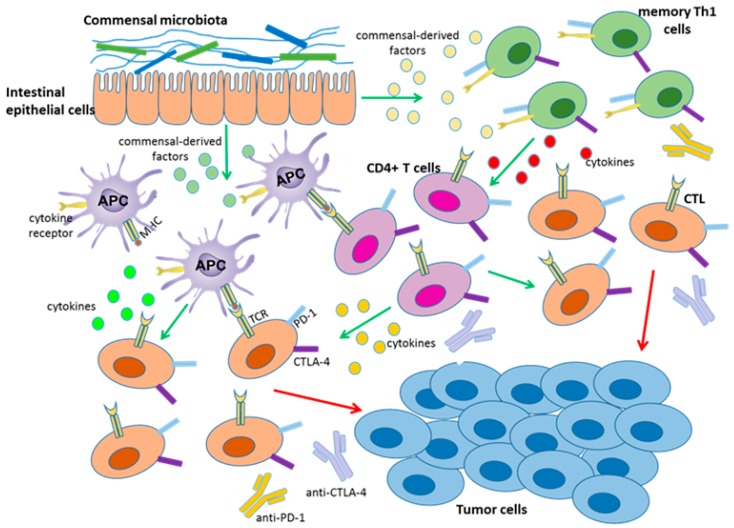
Certain gut microbiota or soluble bacterial products can enhance the efficacy of CTLA-4 and PD-1 checkpoint blockade. Gut bacteria promote the activation and maturation of DCs (APC) most likely by secreting commensal-derived factors, then presenting tumor antigens to prime and support antitumor T cell (CTL, CD4^+^ T cells) responses. Gut bacteria can also activate memory Th1 cells through secreting commensal-derived factors, up-regulating IFN-γ and IL-12 production and down-regulating IL-10 expression to maintain immune activation. The activity and function of tumor-specific T cells can then be enhanced by anti-PD-L1 therapy or anti-CTLA-4 therapy.

As mentioned above, PD-1 and CTLA-4 blockade therapies demonstrate more effective outcomes in patients with high antitumor immunity [3,8]. However, only some patients develop such acute immune responses, which may be associated with the commensal microbiota. In 2015, Sivan et al. found that *Bifidobacterium* was associated with antitumor effects in mice combined with anti-PD-1 therapy. In this study, antitumor CTL responses were compared in genetically similar C57BL/6 mice with melanoma derived from Taconic Farms (TAC) and Jackson Laboratory (JAX), harboring distinct commensal microbiota. The TAC mice generated more aggressive tumors than the JAX mice, and the JAX mice presented increased CD8^+^ T cell tumor infiltration. Then, researchers transferred feces from the JAX mice to the TAC mice, and the CTL responses were restored and the tumor burden was reduced in the TAC mice. Additionally, combination treatment with PD-1 blockade therapy nearly abolished tumor outgrowth in TAC mice. Furthermore, 16S ribosomal RNA sequencing demonstrated that *Bifidobacterium* harbored in JAX mice was associated with the enhanced tumor control and the synergistic effects with the anti-PD-1 therapy [58]. In addition, another study showed that the antitumor efficacy of CTLA-4 blockade therapy was associated with *B. thetaiotaomicron* or *B. fragilis*, some of which were likely to be specific targets of T cell responses. Moreover, fecal microbial transplantation from humans to germ-free mice favored the outgrowth of *B. fragilis* with anticancer properties in melanoma-bearing mice treated with CTLA-4 antibodies, demonstrating that these bacteria can synergize with checkpoint blockade therapy [59]. Together, these findings suggested that particular gut bacteria can enhance anti-PD-1 and anti-CTLA-4 therapy through different mechanisms (Figure 1). On the one hand, *Bifidobacterium* and *B. fragilis* enhanced intratumoral DC activation and CTL responses in tumor-draining lymph nodes, thus driving antitumor immunity. Intriguingly, this enhancement was not related with microbiota transfer to extra-intestinal sites. It may be that commensal-derived factors promote DC activation and soluble systemic chemokines recruit DCs to the TME [58,59]. On the other hand, memory T-cell responses were enhanced in mice with specific immunomodulatory species during CTLA-4 blockade. Previous studies observed memory Th1 responses and IFN-γ production, with little concomitant IL-10 production, in *B. fragilis* and *B. thetaiotaomicron* specific mice treated with a CTLA-4 mAb [60,61]. However, how these microbiota enhance antitumor immune responses in distant sites remains unknown. Therefore, investigating key mechanisms may be crucial for gut microflora to serve as immunotherapy adjuvants to augment checkpoint blockade therapy.

### 2.5. Mutual Regulation among Immune Checkpoints

Although breakthroughs in immune checkpoint blockade therapy have been achieved in recent years, in many cases, the blockade of a single checkpoint (CTLA-4, PD-1, TIGIT, etc.) cannot achieve the desired therapeutic effects [62,63], which may be related to mutual modulation by multiple immune checkpoint receptors or ligands in the tumor microenvironment. Here, we discuss the interactions between PD-1, TIGIT, PVR, CD226, CD96 and CD112 and focus on how they work together to deliver immune stimulatory or inhibitory signals among tumor cells and immune cells within the TME. 

**Figure 2:**
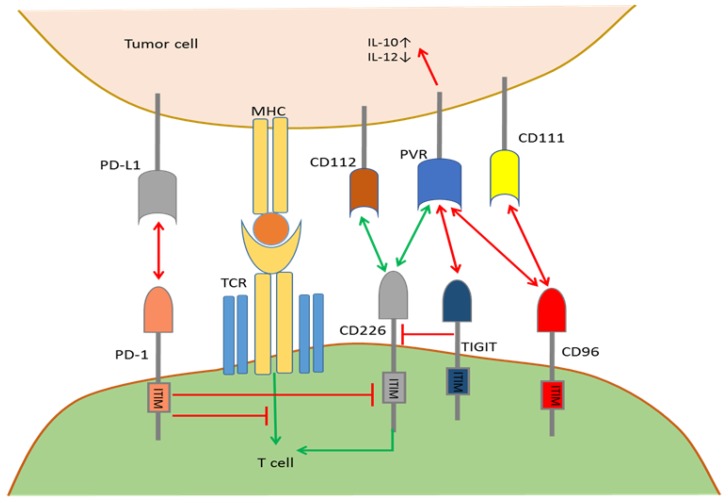
Mutual regulation by the PD-1/CD226/TIGIT/CD96 pathways. TIGIT and CD96 bind to PVR with a higher affinity than CD226 and outcompete CD226 for binding to inhibit T cell activation. TIGIT triggering of PVR induces an inhibitory signal into tumor cells, which up-regulates IL-10 and down-regulates IL-12. TIGIT interferes with CD226 homodimerization in cis and prevents CD226/PVR engagement. Whether TIGIT can directly deliver co-inhibitory signals in T cells after binding to PVR remains unclear. CD226 binds to CD112 and PVR to deliver co-stimulatory signals. CD96 binds to CD111 and PVR to deliver co-inhibitory signals. PD-1 binds to PD-L1 and interferes with CD226 signal transduction to maintain immune resistance in the TME.

TIGIT is expressed on αβ T cells, memory T cells, Tregs, follicular helper T cells (TFH), NKT cells and NK cells, mostly upon T cell activation [64,65]. CD96 expression is limited to immune T cells, including αβ and γδ T cells and NKT cells [66]. Conversely, PVR (CD155), CD112 and CD226 (DNAM-1) are expressed by dendritic cells (DCs), T cells, tumor cells and many other cell types. PD-1 is only expressed by T cells upon priming or activation. Within the TME, CD226, TIGIT, and CD96 bind to PVR with strikingly different affinities (Figure 2). First, the TIGIT interaction with PVR is of a much higher affinity compared to the TIGIT interaction with CD112, participating in the negative regulation of activated T cells and NK cells by up-regulating IL-10 expression and down-regulating IL-12 expression in tumor cells or APCs [67]. However, there is no strong evidence that TIGIT can inhibit T cell activation directly through signal transduction after binding to PVR. Second, TIGIT can directly bind to CD226, which is an immunocyte co-stimulatory molecule on the same T cells, thus blocking the cis-homodimerization of CD226 [63]. Therefore, TIGIT can indirectly inhibit the CD226-transduced immune co-stimulatory signals, but whether cis-homodimerization of CD226 is required for signaling remains unclear. Third, PVR, which is a ligand for the co-stimulatory molecule CD226, has a much greater affinity to TIGIT than CD226 so that TIGIT can block PVR/CD226 pathway-mediated immune co-stimulation by competitively binding to PVR, which is analogous to the higher affinity of CTLA-4 for binding B7 ligands than CD28 [68]. Additionally, studies by Joe-Marc Chauvin et al. revealed that PD-1 and TIGIT are both overexpressed on the vast majority of tumor antigen-specific circulating CD8^+^ T cells and tumor infiltrating CD8^+^ T cells. Compared to blocking PD-1 alone, combined blockade of both TIGIT and PD-1 can significantly enhance antitumor function of CD8^+^ T cells, which depends on CD226 signal transduction [69]. Fourth, human CD96 selectively binds PVR with an affinity between TIGIT and CD226, while CD96 binding to nectin 1 (CD111) is only observed in mouse models [65,70], delivering immune suppressive signals. Although whether CD96 can form a signaling-competent homodimer or is capable of heterodimerization with TIGIT or CD226 is unknown, such modulations can alter ligand binding affinities among PVR/TIGIT/CD226 and affect the PVR/TIGIT/CD226 plasma membrane residence time or intrinsic signal transduction pathways within the TME. In all, structural circumstances and dynamic modulation of the co-expression of CD96, TIGIT, CD226, PVR, and PD-1 and their differential affinities for ligands codetermine whether the final immune response in the TME will be stimulative or suppressive. The combined specific checkpoint blockade can be a promising direction for personalized tumor medicine.

## 3. Future Directions for Personalized Cancer Immunotherapy

Cancer treatment has always been a worldwide challenge, largely due to different immune circumstances among patients. Tumor development in the TME among individuals, together with different levels of expression of immune checkpoint modulators, determines the complexity of cancer therapy. Although immune checkpoint blockade therapy has shown unprecedented clinical efficacy, cancer progression cannot be controlled in the majority of patients, and the safety and toxicity of checkpoint blockade drugs also requires clinical management. Reasons may be that tumor cells can evade immunomediated recognition through new pathways and PD-1 and CTLA-4 blockade can also cause up-regulation of other immune inhibitory receptors like VISTA and TIM-3 [71,72]. In 2015, studies stratified the TME into four types based on PD-L1 expression (positive or negative) and the presence or absence of TIL in melanoma patients (Type 1: PD-L1^+^, TILs^+^; Type 2: PD-L1^−^, TILs^−^; Type 3: PD-L1^+^, TIL^−^; Type 4: PD-L1^−^, TILs^+^) [73,74,75]. This stratification partly explains the variability of checkpoint blockade efficacy among patients and builds a model to understand how to better tailor combined treatments to the TME. In addition, most recently, a study from Zurich University found that before receiving PD-1 inhibitor therapy, the number of CD14^+^CD16^−^HLA-DR^hi^ monocytes in the patient’s blood is the most accurate indicator of progression-free survival and overall survival in patients. This finding explains why PD-1 blockade therapy is only effective for a small number of patients and indicates that detecting the proportion of monocytes in peripheral blood mononuclear cell components may be a biomarker for predicting patient response to PD-1 inhibitor therapy [76].

As mentioned above, immune checkpoints in the TME are modulated by myeloid and lymphoid cells, oncolytic virus, epigenetic modulators, gut microbiota and co-expressed checkpoints. However, the degrees of regulation and which regulations are dominant differ for each patient, meaning that therapies targeting specific checkpoint modulation pathways need to be applied to specific patients. First, DNA methyltransferase and HDAC inhibitors (including 5-azacitidine (Vidaza), 5-aza-2′-deoxyazacytidine (decitabine)) can inhibit MDSCs with much lower doses than required for suppressing tumor cells within the TME, representing a better treatment for patients with high MDSC population in the TME [20,77]. Second, OX-40 is up-regulated after Treg activation and GITR is continuously expressed on Tregs, both of which are co-stimulatory receptors belonging to the TNF receptor superfamily. Cancer patients with high levels of activated Tregs can be treated with anti-GITR and anti-OX-40 agonistic antibodies, combined with PD-L1 blockade [78,79]. Third, vaccines made of oncolytic viruses, such as adenovirus, Maraba virus and vaccinia virus, display both oncolytic activity and a remarkable ability to promote adaptive antitumor immunity in patients with low lymphocyte infiltration rates within the TME [80,81]. Fourth, implanting immunogenic gut microbiota (*B. fragilis*, *B. thetaiotaomicron*, etc.) into specific patients can formulate live commensals to reinstate anticancer adaptive T-cell responses against tumor cells and facilitate anti-checkpoint efficacy [58,59,82]. Although attempts to target checkpoint modulation are still under clinical trials and investigation, these methods provide a direction for the future development of personalized cancer treatment, making immune checkpoint blockade therapy a powerful weapon for fighting cancer.

## Figures and Tables

**Table 1:** Oncolytic viruses in combination with immune checkpoint blockade.

Viruses	Target Checkpoints	Modifications	Cancers Selected for Clinical Trials
Measles virus (MV)	CTLA-4, PD-1	α-PD-1 and α-CTLA-4 encoding	Melanoma, lymphoma, hepatocellular carcinoma, ovarian cancer, myeloma
Herpes virus (HSV)	CTLA-4, PD-1	GM-CSF encoding; Ipilimumab combination; ICP34.5 deletion	Melanoma, head and neck cancer, pancreatic cancer, breast cancer, glioblastoma
Adenoviruses (Ad)	CTLA-4, PD-1, 4-1BB, PVR	GM-CSF, IL-2, IL-12, TNF-a encoding; E1B deletion; a-PD-1, a-CTLA-4,4-1BBL encoding	Head and neck cancer, pancreatic cancer, ovarian cancer, colorectal cancer, melanoma, glioblastoma
Reovirus	PD-1	None	Glioma, sarcomas, colorectal cancer, non-small cell lung cancer (NSCLC), ovarian cancer, melanoma, pancreatic cancer, head and neck cancer
Coxsackievirus	CTLA-4, PD-1	None	Melanoma, breast cancer, and prostate cancer
Newcastle disease virus (NDV)	CTLA-4, PD-1, ICOS	GM-CSF, IL-2, EGFP encoding	Colorectal cancer, hepatoma, lung cancer, prostate cancer
Vaccinia virus (VV)	CTLA-4, PD-1	GM-CSF, IL-10, VEGF encoding	Melanoma, liver cancer, colorectal cancer, breast cancer, and hepatocellular carcinoma, pancreatic cancer

Cancers listed in Table 1 refer to cancer types which were selected for clinical trials on patients and phaseI, phaseII and phaseIII clinical trials were included. Most clinically relevant oncolytic viruses utilize attenuated vectors or naturally occurring less virulent variants of particular viruses in order to prevent acute and long-term toxicity.
